# Ketogenic enteral nutrition as a treatment for obesity: short term and long term results from 19,000 patients

**DOI:** 10.1186/1743-7075-9-96

**Published:** 2012-10-30

**Authors:** Gianfranco Cappello, Antonella Franceschelli, Annalisa Cappello, Paolo De Luca

**Affiliations:** 1 Clinical Nutrition Service of the Department of Surgery Paride Stefanini, University of Rome La Sapienza, Rome, Italy

**Keywords:** Obesity, Enteral nutrition, Ketogenic nutrition, Body composition

## Abstract

**Background:**

Only protein diet has been used successfully to prevent loss of lean body mass first in post-surgical and then in obese patients. We studied overweight and obese patients receiving short treatments of an exclusively protein-based nutritional solution as 24-hour enteral infusion.

**Methods:**

19,036 patients (age 44.3 ± 13, M:F = 2:5) with an initial body mass index of 36.5 ± 7.1 underwent 10-day cycles of enteral nutrition through a fine nasogastric tube. The nutritional solution consisted solely of 50–65 g of proteins, plus vitamins and electrolytes. The 24-hour infusion was controlled with a small portable pump. Before and after each 10-day cycle body composition was checked with a Handy 3000 impedance analyzer. At the onset of treatment, average fat mass was 40.9 ± 12.8 kg while body cell mass was 42.7 ± 7.2 kg in males and 27.4 ± 4.6 kg in females.

**Results:**

After an average of 2.5 cycles the patients lost 10.2 ± 7.0 kg of body weight, 5.8 ± 5.5 kg of fat mass and 2.2 ± 3.3 kg of body cell mass. No significant adverse effects were recorded except asthenia and constipation which were easily controlled with therapy. Long-term results were obtained from 15,444 patients and after an average of 362 ± 296 days we found a mean weight regain of 15.4%.

**Conclusion:**

Ketogenic Enteral Nutrition treatment of over 19,000 patients induced a rapid 10% weight loss, 57% of which was Fat Mass. No significant adverse effects were found. The treatment is safe, fast, inexpensive and has good one-year results for weight maintenance.

## Background

Obesity is a 21st century epidemic, and its relentless spreading is due to many different reasons. One reason is that the classic treatment, a long term hypocaloric diet, is unsuited to the spirit of our century that always aims for fast results. Dieters want instant weight loss and do not want to lose “only” a few pounds each week. Finding a fast and safe weight loss treatment could be the crucial battle to win in the war on obesity.

We know that weight loss is a consequence of negative caloric balance and the more negative the caloric balance the more rapid the weight reduction will be. Given this logic, total fasting should be the most rapid way to lose weight but it is impractical for two reasons:

1. It would cause extreme hunger [1].

2. It would entail loss of lean body mass (LBM), which can be unsafe for the patient [2]. Total fasting also causes neutropenia [3], lowers renal creatinine clearence [4] and increases levels of serum bilirubin [5]. The total nitrogen loss after 3 to 4 weeks of total starvation would be approximately 200 g, corresponding to 1,250 g of protein and equivalent to a loss of some 6 kg of muscle tissue [6,7]. The obese patient will lose body mass in the wrong places (e.g. thighs, limbs, chest) generating a cachectic appearance. Furthermore he will rapidly regain all the lost weight as the body works to rebuild its LBM [8]. Thus, optimal weight loss must be achieved through reducing fat mass.

An important experiment by Blackburn and colleagues in 1973 [9] demonstrated that, during fasting, a continuous intravenous infusion of an amino acid solution could greatly reduce protein loss. Because the treatment was able to totally prevent loss of LBM during fasting, it was said to have a protein sparing (PS) effect. Blackburn explained the PS effect through the action of insulin, the main regulator of energetic fuels, by reasoning that the infusion of amino acids during fasting reduced insulin levels and therefore prevented muscle catabolism while at the same time a strong lipolytic effect was promoted with high serum ketone bodies (KB) [10]. Increased serum KB will not harm the patient because high serum KB will eventually increase insulin secretion, modulating the lipolytic effect [11,12]. Furthermore high serum KB will reduce or eliminate hunger [13,14].

Blackburn et al. immediately realized the importance of the PS effect for the treatment of obesity. They invented a very low calorie protein diet [15], the protein sparing modified fast (PSMF), but their subsequent studies on obese patients were based on an oral protein diet which had two main drawbacks: (1) glucose intake could not be reduced to zero and (2) protein intake stopped during the night. Normally overnight fasting does not entail muscle catabolism because hepatic glycogen sustains the patient’s energy requirement [16-18], but after one day of a high protein diet glycogen deposits are greatly reduced [16] and overnight fasting will entail muscle catabolism. As a result the lipolytic effect of the PSMF is reduced and many weeks of the diet are needed to obtain a 10% weight loss15.

To add to the concerns about the PSMF, it was reported that the diet was suspected of causing severe cardiac arrhythmias [19-23]. A loss of cardiac protein content was supposed to be the mechanism the cardiac complications [24]. Other researchers [19,25,26] attributed the effect to the low quality of protein used in the PSMF, however the diet is thought to be safe for treatments not longer than 10 days [22,23].

Around the time of the original report published by Blackburn and colleagues, our Nutrition Unit at the University of Rome was using a PS diet (via 24 h IV infusion of strictly amino acids, minerals and vitamins) on our obese surgical patients. While the aim of our treatment was to improve our surgical results, we also noted that we confirmed Blackburn et al.’s findings with regards to weight loss [27].

In the 1980s, when enteral nutrition took the place of parenteral nutrition, we had the same results with a 24 h enteral infusion of a solution of only proteins, electrolytes and vitamins. Those results were not published. Enteral protein sparing was generally used for obese patients who needed nutritional support for postoperative complications or acute pancreatitis. Obesity was never the main indication for the enteral protein sparing treatment. They were all hospitalized patients. Nitrogen balance showed an acceptable level of nitrogen losses and blood samples showed good tolerance of the treatment with normal blood urea and electrolyte levels. No cardiac complications were ever documented.

Six years ago the young, obese daughter of one of our enteral protein sparing patients (with a postoperative colon fistula) asked to be treated only for her obesity. She underwent a home treatment and lost 15 kg of weight. Since then, thousands of patients have come to the University of Rome from all over Italy to be treated and they had all heard of us only by word of mouth. They signed a formal consent and were given a standard apparatus for enteral nutrition (a small nasogastric tube and a portable pump) for a 24 h infusion of protein to be administered in cycles of 10 days.

In a preliminary study we found that the infusion of 50–65 g/day of high biological value protein (whey protein) caused a mild ketonemia (100–120 mg%), eliminated hunger and greatly reduced the loss of lean body mass while patients were losing weight rapidly. We named this approach Ketogenic Enteral Nutrition (KEN).

The aim of this study is to determine the feasibility of KEN therapy for a large population of overweight or obese patients and to assess its clinical results from a retrospective analysis of the records of all patients who underwent at least one cycle of KEN treatment. We also wanted to compare the results from KEN treatment to the results from other PSMF studies in which protein intake was given orally and was suspended during the night. Long term results have been as well investigated.

## Methods

The study was performed at the University La Sapienza in Rome within the Clinical Nutrition Service (CNS) of the Department of Surgery Paride Stefanini and included overweight and obese patients who did not have success with previous dietetic treatments for obesity.

Patients with type 1 diabetes, renal failure, heart failure, hepatic failure, history of severe cardiac arrhythmias, severe eating disorders or who were pregnant or lactating were excluded. Young patients under 14 were included only if their body mass index (BMI) was over 40 and they had undergone a series of dietary treatments without success. The patients were self-referred and each patient had to sign an informed consent release before the beginning of the treatment. The procedures were in accordance with the Helsinki Declaration of 1975 (as revised in 1983).

In a period of 5 years (from 2006 to 2011) 19,036 patients underwent at least one cycle of KEN treatment. The patients’ ages varied from 10 to 78 (average 44.3 years). The male:female ratio was 2:5. The average weight at the start of the treatment was 101.4 kg, and was higher for men than women (118.9 kg vs 94.9 kg, respectively). The mean BMI was 36.5 kg/m2 (in the limit for class 1 and class 2 of obesity) and was also higher in men than women (38.2 vs 35.8, respectively).

Each patient came to the CNS and received a medical checkup before the beginning of treatment and a 50 Hz impedance test for body composition analysis (Handy 3000; DS Medica, Italy). The body composition was calculated by a computerized program which was provided by the manufacturer, according to the three-compartment model [28].

As shown in Table 1 at the start of the treatment men had a very high body cell mass (BCM; 42.7 kg vs 27.4) while the average fat mass (FM) was 40.9 kg and did not differ between men and women (40.0 kg vs 41.7).

**Table 1:** Patients’ baseline data before KEN (n = 19,036)

	**Total**	**M**	**F**
Cases	19,036	5,148	13,888
Age	44.3 ± 13.0	44.7 ± 12.4	44.2 ± 13.2
Initial weight (kg)	101.4 ± 22.9	118.9 ± 22.9	94.9 ± 19.2
BMI (kg/m2)	36.5 ± 7.1	38.2 ± 6.8	35.8 ± 7.1
BCM (kg)	31.6 ± 8.7	42.7 ± 7.2	27.4 ± 4.6
FM (kg)	40.9 ± 12.8	40.0 ± 12.6	41.7 ± 12.8
TBW (kg)	43.5 ± 10.9	57.4 ± 9.2	38.3 ± 5.7

Table 1 shows the average age, weight and body composition of the patients before the start of the KEN treatment. (BCM, body cell mass; FM, fat mass; TBW, total body water).

**Figure 1:**
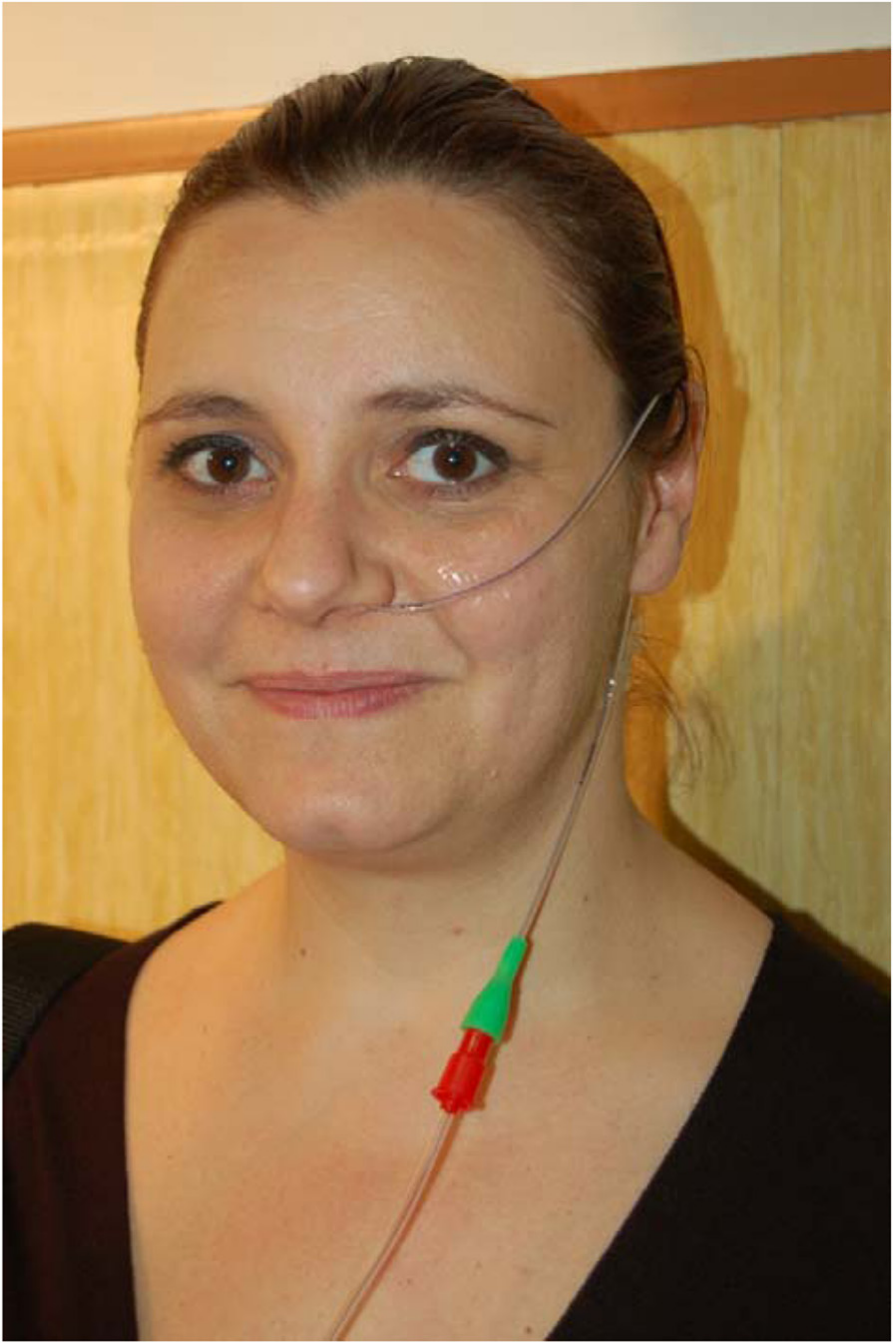
6-French polyurethane tubes are almost invisible and very well tolerated.

A 6-fr polyurethane tube was inserted through the nose (Figure 1). The tube was held in place on the patient’s cheek and ear by transparent tape.

The patients were given an enteral portable pump and a short course to educate them about its use and about the basics of KEN. The infusion rate was regulated to provide, on a continuous, 24 h daily infusion, 2 l of fluid and a dose of 50 g of protein (K1000®, Table 2) for women and 65 g for men (resulting in an average dose of 0.85 g/kg of ideal body weight in women and 0.89 g/kg in men) and 13–17 mEq of potassium.

**Table 2:** (g%) Composition of the nutrition powder (K1000®)

	
Proteins	90.0
Carbohydrates	1.80
Fats	0.80
Calcium	0.40
Potassium	1.00
Phosphorus	0.20
Sodium	0.10
Magnesium	0.05

Table 2 Composition of K1000® (Nutrimed 2000 srl, Italy), the nutrition feed used for the KEN, made of whey proteins enriched with potassium chloride, lecithin and bovine hydrolyzed collagen.

Each patient received, in addition, a supplement providing the RDA [29] for all vitamins and essential minerals. They were also given a 50 g dose of polyethylene glycol to be taken on the first, fourth and seventh days of treatment in order to increase intestinal motility and to make up for the absence of fibers in the protein solution.

The KEN cycles were domiciliary and a medical doctor (MD) was available by telephone 24 h/day.

To ensure continuous ketosis during the treatment, the patients had to stop feeding. They were allowed to drink as much as they wanted, however they were only allowed to drink water, tea, coffee and chamomile tea without sweeteners.

During the cycle the patients were given a diary to monitor weight, feeling of hunger (on a visual scale from 1 to 10) and ketonuria (Ketur test, Roche, Italy).

Hypertensive patients were suggested to stop their usual medication to avoid symptomatic hypotension that was described by other authors during PSMF [30,31]. They had to monitor their blood pressure daily and contact the MD in case of increased blood pressure.

As in other PSMF studies [32], diabetic patients were asked to suspend their usual medications to avoid hypoglycemia. They were also asked to monitor glycemia 3 times a day and to contact the MD in case of blood glucose >160 mg%. Stopping or reducing the medications did not cause any trouble, because KEN treatment rapidly reduces hypertension and hyper-glycaemia. Many times we had to discuss this matter with the physicians of our patients.

Patients affected by gout and/or hyperuricemia were supplied with daily doses of allopurinol.

After each KEN cycle, patients returned to the CNS for tube removal, to report all treatment data (including daily evaluation of hunger level on a 1 to 10 visual scale, body weight and ketonuria) and to undergo another impedance test for body composition evaluation.

Each patient was free to choose the number of KEN cycles he or she wanted to undergo. If the patient wanted to undergo another treatment cycle they were first given a rest period of at least 10 days during which they were advised to follow a low–carbohydrate, normocaloric diet.

They were domiciliary patients and it was impossible to schedule from the start of treatment the number of cycles each patient had to undergo. We just told to each patient his ideal body weight and informed him of the risk of been obese or overweight.

All patients during the 5-year period of this study underwent 1 to 21 cycles of KEN treatment (an average of 2.5 ± 0.03). The total duration of treatment time from the first 10-day KEN cycle to the last, including variable-length rest periods (some patients came for a new cycle years later) lasted from 9 to 1400 days (mean 68 ± 96).

Long term results data were collected by a telephone survey which were assessed by nurses.

## Results

Table 3 shows the final results. The male patients lost a mean of 11.9 kg of weight which corresponded to 9.96% of initial body weight. Women also lost about 10% of initial body weight.

**Table 3:** Body Composition changes after treatment with KEN in male and female patients (*P < 0.0001. Student paired t-test)

**Weight and body composition changes after KEN treatment (n = 19,036)**						
	**Total**	**Variation ± SD**	**M**	**Variation ± SD**	**F**	**Variation ± SD**
Body weight (kg)	91.2	−10.2 ± 7.0*	107.0	−11.9 ± 7.9*	85.4	−9.5 ± 6.5*
BMI (kg/m2)	32.8	−3.7 ± 2.5*	34.3	−3.9 ± 2.6*	32.2	−3.6 ± 2.5*
BCM (kg)	29.4	−2.2* ± 3.3*	39.7	−3.0 ± 4.2*	25.6	−1.8 ± 2.8*
TBW (kg)	40.1	−3.4* ± 2.8*	52.7	−4.7 ± 3.5*	35.3	−3.0 ± 3.4*
FM (kg)	35.1	−5.8* ± 5.5*	34.3	−5.7 ± 6.0*	35.9	−5.8 ± 5.2*

The body composition analysis performed before and after treatment showed that 57% of weight loss was came from FM and 22% came from BCM.

No technical complications were reported during the introduction of over 50,000 nasogastric tubes in a 5-year period; neither were there any metabolic complications due to ketonemia or electrolyte imbalances.

Ketonuria was observed in most patients by the 2nd day of the KEN treatment and increased within 2–3 days to 100–120 mg%. Commonly the ketonuria was accompanied by moderate halitosis, however this did not prevent the patients from carrying out normal daily activities.

By the 5th day of treatment 24% of patients reported a strong sense of asthenia, even if they had normal blood pressure levels. By the end of the treatment, 12% of patients reported a mild sense of hunger.

Nearly one quarter (22%) of the patients were diabetic and receiving treatment for their condition at the start of treatment. As was observed by other authors [32], 92% of the diabetics in our study also had to suspend their medications during the treatment periods because the lack of carbohydrate in the nutritional solution was sufficient to lower their glycaemia on its own. Of these patients who suspended anti-diabetic therapy, 22% reported glucose values as low as 60 mg/dL. However, no cases of clinical hypoglycemia were reported neither was glucose supplementation needed.

Reduced hypertension during PSMF has been reported previously [30,31]. Similarly, in our study only 20% of the hypertensive patients who had suspended their medications during the treatments needed to resume their medications afterwards, and even then they needed a lower dose.

Complications of KEN:

1. Asthenia 24%

2. Mild sense of hunger 12%

3. Constipation (need to increase Macrogol) 5%

4. Problems with the pump 4%

5. Damage of the external part of the tube (e.g. shaving) 2%

6. Gastric hypersecretion 2%

7. Nausea and vomiting 1%

8. Intolerance to the nasal tube 0.03%

9. Ulcerations or bleeding due to the tube not observed

10. Breakage of the tube in esophagus or in the stomach not observed

11. Perforation or bleeding of the stomach not observed

### Long term results

After the last KEN cycle (after 31–1882 days, average 408 ± 309 days) 15,444 patients could be reached by telephone. We found that 55 patients had undergone bariatric surgery, which is not surprising since many patients had been referred to us for preparation prior to bariatric surgery because a preoperative loss of weight causes an improvement of cardiovascular and thromboembolic risk factors [33]. Regardless these individuals were excluded from the survey. All the other patients had regained an average of 1.57 ± 7.15 kg of body weight (15.4% of the mean weight loss). 38.9% of patients presented a variation of ±3% and we can consider that they maintained their weight loss, 36.9% gained weight (sometimes to a value higher than the weight they had before the KEN treatment) while 24.1% lost more weight because they were able to return to their sporting activities.

## Discussion

It is insightful to compare the results of this study with other PSMF studies in which protein was given by mouth. In one study [15], weight loss after 17 weeks of treatment was 21 kg versus 10.2 kg in 3.5 weeks in the present study. Another smaller study [34] found results are closer to ours: 15 patients lost 14.4 kg after 6 weeks of PSMF with lactalbumin-derived protein dosed at 60 g/day. In a third study [35], patients lost around 8 kg in 4 weeks on either a lean meat, fish, and fowl diet of 450 kcal or an isocaloric high-protein liquid diet. None of the studies reported any significant clinical complications. The last study [35] found that the liquid formula diet was less palatable than the whole food diet. In contrast we found that our patients were able to lose more weight in less time, likely due to the fact that using a 24 h infusion we could further reduce protein (caloric) intake while sparing lean mass.

The KEN diet is a well tolerated treatment that produces very rapid weight loss and gives the patient a psychological boost because he/she sees immediate results. This enthusiasm gives the patient the resolve to continue with the treatment. Remember the thousands of patients that came to us to undergo treatment were not responding to any advertisements, rather they had heard of us by word of mouth.

Another benefit of the KEN treatment is that it is a low-cost treatment. Investments in the procedure include a 3-h course covering the principles of the treatment to new patients, scales, stadiometers, impedance apparati and pumps are relatively inexpensive. Therefore, as all patients stop eating for 10 days, we can say to them that the treatment will cost about the same amount as eating their normal diet for 10 days.

A 6-fr nasogastric tube is very well tolerated; patients get used to sensation within 10 minutes after insertion and no longer feel its presence. Only a very small percentage of patients (0.03%) decided to stop treatment when the tube was placed through the nose. In no cases did the tube cause ulceration or bleeding, nor was there any breakage in the esophagus or stomach. However, in some cases the external part of the tube was damaged by the patients themselves when trying to shave or when trying to replace the tape using scissors. All these problems were solved by replacing the tube itself.

We have assumed that the nasal intubation and the pump are essential to the success of our treatment for controlling the intake of proteins during both day and night and for reducing the catabolism of lean mass. While this assumption should be tested in a double blind study, our results still show that the KEN diet is more effective in promoting weight loss than the PSMF diet and reducing the length of treatment to 10 days prevents the risk of cardiac complications [19-23].

Prof. Jay Mirtallo, President of the American Society for Parenteral and Enteral Nutrition, recently wrote a letter to the New York Times [36] about the nasogastric tube for KEN treatment saying that “to report on someone using this medical therapy as a weight loss method detracts from the health benefit achieved by patients with very severe diseases”. We agree with him-- enteral nutrition therapy is normally used to feed malnourished patients who are unable to eat food by mouth for various reasons (e.g. dysphagia, cancer) and its utilization has a very important therapeutic value. However, in our experience, extending the use of the nasal tube to obese patients did not in any way impair the use of enteral therapy in malnourished patients. To the contrary, in Italy we noticed that, after thousands of patients began asking for the therapy as a treatment for obesity, the number of malnourished patients asking for the tube as a life support doubled. We think this is likely because they were able to observe how a high quality of life can be maintained during the treatment, and that it is not a big deal to have a small tube in your nose. Furthermore we think the application of enteral therapy in the obese opens new possibilities in healthcare, as worldwide there are the millions of obese patients who could benefit from this treatment, vastly outnumbering the thousands of people with cancer or neurologic dysfunction that require a nasal tube.

With regard to the complications of KEN treatment including asthenia and mild lightheadedness, which have also been reported with the PSMF diet [15], the symptoms were easily relieved by increasing salt intake [15].

Gastric hypersecretion was present as acid reflux or pyrosis in 2% of patients during KEN. This effect could be due to the protein infusion [37,38] or it could be connected with the mild metabolic acidosis [39] that occurs in ketonemic diets [40]. Constipation is also commonly reported during ketonemic diets [15,40], probably due to the lack of fibers in the protein solution. This issue can be resolved by increasing polyethylene glycol (PEG) administration.

Nausea and vomiting has been reported also in another study [15], and they are probably in response to the gastric hypersecretion caused by the ketonemia or by rapid intake of PEG. It is a rare complication but concerning because it can lead to expulsion of the nasal tube which then has to be re-inserted.

KEN treatment is not an option for long-term dieting because it contains 0% carbohydrates and 0% lipids. Rather, KEN is suitable as a 10-day controlled period of starvation during which the protein sparing effect of the continuous infusion of protein allows a fast and safe reduction of weight. Although the weight loss is still 22% BCM, this is to be expected given that the obese and overweight patients show elevated BCM before the start of treatment. This has been confirmed by other authors [41] and a minor loss of BCM is not significant. In future studies we will modulate the protein infusion with respect to the impedance analysis in the aim of reducing BCM loss and of increasing FM loss from their current levels.

Losing 4 kg of fat mass means burning 36,000 calories in 10 days. In other words, to lose 1 kg of fat an individual would need to walk for 75 km42. We tell our patients to live an active life, to maintain their normal daily activities in spite of the presence of the nasal tube and to walk at least one hour per day (if they can). This probably is not enough to burn all those calories. But we must take into account other outputs, such as the loss of ketones in the urine, in the breath and in the sweat; that is, during ketosis there is some insensible loss of calories. Furthermore protein infusions are reported to increase energy expenditure by increasing thermogenesis [42-44].

The long term results from our study were very positive. The data were collected by a telephone survey, and while we could not check the patients’ weights directly, we feel the self-reported body weights are accurate and other reports on long term results after weight loss treatments are also based on telephone surveys [45]. At the end of a patient’s final KEN cycle they were advised to regularly check their body weight and were given advice on how to reduce their weight again if it began to go back up. They were also advised to consult an MD as needed, however it was rare that they asked for help. In our study we observed a 15.4% weight regain after one year, which is an excellent result if we compare it with other reviews in which a 30-35% weight regain after one year is reported [45]. This difference may be because KEN treatment spares free fat mass, and this reduces weight regain [46]. Furthermore the initial weight of our patients was higher than in most weight maintenance studies [47] which may also account for our improved long-term results [48,49].

Weight loss in our diabetic and hypertensive patients also promoted long-term improvement to their conditions, as has been reported in another study [50], and these results will be the subject of a separate report.

Limitations of this study are related to (1) the lack of a control group, which would be impossible to obtain in our settings. (2) We could not plan the treatment of each patient at the start because they were on domiciliary treatment and (3) many patients come back for a new cycle after years of rest making uncertain the evaluation of their overall clinical outcome.”

## Conclusions

During the course of 5 years nearly 50,000 small 6-French nasogastric tubes were inserted without complications and they were well tolerated by our patients. This study demonstrates that 10-day KEN cycles can induce rapid weight loss; in a very large number of patients we easily obtained a 10% weight loss, 57% of which was FM. No significant adverse effects were found. On a 1-month/5-year follow up, mean weight regain was 15.4%. The KEN cycles are suitable for normal living and the cost is minimal, indeed some patients spend less on the treatment than they would spend on 10 days worth of food. Ultimately we conclude that KEN treatment is safe, fast, inexpensive and has good one-year results for weight maintenance. We propose that KEN is a new approach to obesity treatment which is faster and more effective than hypocaloric diets [47], without the complications of bariatric surgery.

## Abbreviations

BCM: Body Cell Mass; BMI: Body Mass Index; CNS: Clinical Nutrition Service; FM: Fat Mass; KB: Ketone Bodies; KEN: Ketogenic Enteral Nutrition; LBM: Lean Body Mass; MD: Medical Doctor; PEG: Polyethilene glycol; PS: Protein Sparing; PSMF: Protein Sparing Modified Fast; RDA: Recommended Daily Allowance; TBW: Total Body Water.

## Competing interests

The authors declare that they have no competing interests.

## Authors’ contributions

GC conceived of the study. GC and AF participated in the design of the study, performed the statistical analysis and have been involved in drafting the manuscript and revising it critically for important intellectual content. AC and PDL carried out the acquisition, the analysis and interpretation of data. All authors read and approved the final manuscript.

## Clinical relevancy statement

Dieters of 21st century want instant weight loss and do not want to lose “only” a few pounds each week. Finding a fast and safe weight loss treatment could be the crucial battle to win in the war on obesity. Ketogenic Enteral Nutrition is a modified approach that allows a fast weight loss. It was tested on thousands of patients and it turned out to be safe and inexpensive, we can say to our patients that the treatment will cost about the same amount as eating their normal diet for 10 days.
